# Inhibition of lncRNA LINC00461/miR-216a/aquaporin 4 pathway suppresses cell proliferation, migration, invasion, and chemoresistance in glioma

**DOI:** 10.1515/biol-2020-0048

**Published:** 2020-07-27

**Authors:** Yanguo Peng, Wangchun Wu, Zhanfang Shang, Wei Li, Shuiyu Chen

**Affiliations:** Department of neurosurgery, The Affiliated Mindong Hospital of Fujian Medical University, No. 89 Heshan Road, Fuan 355000, Fujian, China

**Keywords:** LINC00461, miR-216a, aquaporin 4 (AQP4), glioma

## Abstract

Long noncoding RNA (lncRNA) LINC00461 (LINC00461) is reported to be related to glioma progression. However, the mechanism of LINC00461 in glioma remains unclear. Expression of LINC00461, miRNA (miR)-216a, and aquaporin 4 (AQP4) was detected using real-time quantitative PCR (RT-qPCR) and western blotting. Proliferation, temozolomide (TMZ) resistance, migration, and invasion were assessed by MTT, colony formation, and transwell assays, respectively. The target binding among miR-216a, LINC00461, and AQP4 was confirmed by the luciferase reporter assay. The tumor growth was monitored in the xenograft experiment. LINC00461 was upregulated, and miR-216a was downregulated in glioma tissues and cells, and LINC00461 upregulation was correlated with large tumor size, higher WHO grade and recurrence, and poor overall survival. LINC00461 knockdown suppressed cell viability, abilities of cell cloning and migration and invasion, and TMZ resistance in glioma. Mechanically, LINC00461 was confirmed to sponge miR-216a to affect AQP4 expression. Rescue assays verified that miR-216a downregulation or AQP4 upregulation abrogated the inhibitory effect of LINC00461 knockdown on cell proliferation, migration, invasion, and TMZ resistance *in vitro*. Moreover, LINC00461 downregulation blocked the glioma tumor growth *in vivo.* In conclusion, LINC00461 knockdown inhibits glioma cell proliferation, migration, invasion, and TMZ resistance through miR-216a/AQP4 axis, suggesting LINC00461 as an oncogene in glioma progression.

## 1 Introduction

Human glioma is one of the most prevalent and aggressive malignant tumors in the human central nervous system with high mortality worldwide [1]. It is known that glioma arises from astrocytes or astroglial precursors [2]. According to the histopathology, the World Health Organization (WHO) has divided glioma into four grades: I–IV [3]. The prognosis of glioma remains poor, and the 5-year survival rate of glioma patients in low grade (grades I–II) is 30–70%, while the median survival time of glioblastoma multiform (grade IV) is less than 12 months [4]. Moreover, the rates of recurrence and mortality are still high because of unrestricted proliferation and extensive metastasis of glioma tumors [5]. Therefore, it is imperative to focus on the special molecular mechanisms of glioma tumorigenesis and progression.

Numerous studies indicate that noncoding RNAs including long noncoding RNA (lncRNAs) and small noncoding RNA (microRNAs [miRNAs]) have critical functions across biological processes that regulate glioma initiation and progression [6]. LncRNAs participate in almost all biological processes of different tumors even though they have little protein coding capacity. Aberrant expression of lncRNAs has been discovered in glioma, and thus, these lncRNAs are supposed to be biomarkers and therapeutic targets for the treatment of glioma [1,7].

LINC00461 is a novel cancer-related lncRNA, transcribed from a gene located at an intergenic region of human chromosome 5 [8]. Previously, LINC00461 was found to serve as oncogene and promote progression of different malignant tumors, such as hepatocellular carcinoma [9], multiple myeloma [10], breast cancer [11], and glioma [8,12] as well. In glioma, LINC00461 was dominantly located in the cytoplasm and was highly expressed in glioma tissues [12]. Even though the importance of LINC00461 dysregulation in the proliferation, migration, and invasion of glioma cells has been announced [8,12], the role of LINC00461 in gliomagenesis remains to be fully investigated, as well as its underlying mechanisms.

It is well documented that lncRNAs function as competing endogenous RNAs (ceRNAs), sponging miRNAs to participate in the occurrence and the development of human tumors, including glioma [7]. In this study, the abnormal expression of LINC00461 in glioma tissues and cells was confirmed, as well as the correlation between LINC00461 expression level and clinical features and cumulative 5-year survival. Loss-of-function experiments were carried out to figure out the role of LINC00461 in glioma cell proliferation, migration, invasion, and temozolomide (TMZ) resistance *in vitro* and tumor growth *in vivo*. Mechanically, the LINC00461/miRNA/message RNA (mRNA) pathway was further detected. Our work demonstrated a novel mechanism of LINC00461 as oncogene in glioma progression.

## 2 Materials and methods

### 2.1 Clinical samples and tissue acquirement

Fifty patients with glioma were recruited in this study in The Affiliated Mindong Hospital of Fujian Medical University from year 2013 to 2018. The paired glioma tissues and the adjacent peritumoral brain edema tissues were obtained from patients undergoing surgery. All tissues were directly preserved in liquid nitrogen and stored at −80°C. Glioma samples consist of grades I–II (*n* = 11) and grades III–IV (*n* = 39), which were histologically verified based on the WHO-2007 classification. No enrolled patients in our study received chemotherapy or radiotherapy before surgery. After gross total resection, the patients were followed up by standard-of-care radiation therapy and chemotherapy. The information of patients with glioma was provided in Table 1.

**Table 1:** Clinical characteristics of patients with glioma in the study

Characteristics	*n*	LINC00461		*P*
		High	Low	
Gender				
Female	17	6	11	0.197
Male	33	18	15	
Age (years)				
≥45	37	20	17	0.148
<45	13	4	9	
Tumor size (cm)				
≥3	35	21	14	0.006^a^
<3	15	3	12	
WHO grade				
I–II	11	2	9	0.025^a^
III–IV	39	22	17	
Necrosis				
Yes	8	2	6	0.155
No	42	22	20	
Recurrence				
Yes	21	16	5	0.001^a^
No	29	8	21	

a *p* < 0.05.


**Informed consent:** Informed consent has been obtained from all individuals included in this study.
**Ethical approval:** The research related to human use has been complied with all the relevant national regulations, institutional policies, and in accordance with the tenets of the Helsinki Declaration and has been approved by the Ethics Committee of The Affiliated Mindong Hospital of Fujian Medical University.

### 2.2 Cells and cell culture

Human glioblastoma cells U251 (ECACC, 09063001), A172 (CRL-1620), T98G (CRL-1690), HS683 (HTB-138), and U138 (HTB-16) were purchased from American Type Culture Collection (ATCC; Manassas, VA, USA) and European Collection of Authenticated Cell Cultures (ECACC; Public Health England, Porton Down, Salisbury, UK). The normal human astrocytes (NHAs) were purchased from Type Culture Collection of the Chinese Academy of Sciences (Shanghai, China). All cells were cultured and passaged for 3 days in Roswell Park Memorial Institute 1640 medium (RPMI-1640; Gibco, Grand Island, NY, USA) supplemented with 10% (v/v) fetal bovine serum (FBS; Invitrogen, Carlsbad, CA, USA) in a humidified incubator at 37°C in 5% CO_2_.

### 2.3 TMZ treatment

TMZ was purchased from Sigma-Aldrich (St. Louis, MO, USA), and its stock solution was 100 mM in dimethyl sulfoxide (DMSO; Sigma-Aldrich) in −20°C. Different doses of TMZ (0, 7.5, 15, 30, 60, 120, 240, and 480 µM) were added into medium for 48 h.

### 2.4 Cell transfection

U251 and A172 cells were seeded into 6-well plate (Corning, NY, USA) and 96-well plate (Corning) and incubated overnight. When cells meet 80% confluence, transient transfection was carried out with Lipofectamine™ 2000 (Invitrogen) according to the manufacturer’s instruction. The pcDNA3.1 vector was purchased from Thermo Fisher Scientific (Waltham, MA, USA). The recombinant vectors pcDNA-LINC00461 (LINC00461) and pcDNA-AQP4 (AQP4) were constructed, and the empty vector served as a control. Special siRNAs against human LINC00461 (si-LINC00461#1, 2 and 3), siRNA against AQP4 (si-AQP4), miR-216a mimics, miR-216a inhibitor, and the negative controls were obtained from GenePharma (Shanghai, China). Uniformly, 100 nM oligonucleotides or 2 µg plasmids were used for transfection. As for rescue assays, 50 nM oligonucleotides were co-transfected into U251 and A172 cells. Transfected cells were incubated for 48 h for further study. Sequences of siRNAs were as follows: si-LINC00461#1: 5′-GGAAATGAAAGTGACATTTAC-3′; si-LINC00461#2: 5′-GGAAGCTACTGAAGCAGAAAG-3′; si-LINC00461#3: 5′-CAGCATCAAAATCGAATAATA-3′; si-AQP4: 5′-GCTCAATAGCTTTAGCAATTG-3′; and si-NC: 5′-CCTAAGGTTAAGTCGCCCTCG-3′.

### 2.5 RNA extraction and real-time quantitative PCR

Total RNA in tissues and cells was isolated with TRIzol (Invitrogen). Two hundred nanograms of total RNA was used to synthesize the first-strand cDNA with the Reverse transcription kit (Abcam, Cambrige, UK). The quantitative PCR was performed with SYBR Premix Ex Taq Master Mix (TaKaRa, Shiga, Japan) on ABI 7900 real-time PCR system (Promega). GAPDH was used as an internal control to LINC00461 and AQP4 mRNA, and U6 small nuclear RNA (U6) was for mature miR-216a. The relative expression was calculated according to the comparative threshold cycle value (2^−ΔΔCt^) method. The reactions were performed in triplicate for each sample at least three independent runs, and the primers involved are as follows: LINC00461, 5′-GACATTTACGCCACAACCCACG-3′ (sense) and 5′-AGACAGACCCTCAGATTCCCCA-3′ (anti-sense); miR-216a, 5′-ATCCAGTGCGTGTCGTG-3′ (sense) and 5′-TGCTTAATCTCAGCTGGCA-3′ (anti-sense); AQP4, 5′-GTGACAGACCCACAGCAAGG-3′ (sense) and 5′-TCAACTCAACCAAGGAGACCAT-3′ (anti-sense); GAPDH, 5′-AGGTCGGAGTCAACGGATTT-3′ (sense) and 5′-ATCTCGCTCCTGGAAGATGG-3′ (anti-sense); and U6, 5′-GCTTCGGCAGCACATATACTAAAAT-3′ (sense) and 5′-CGCTTCACGAATTTGCGTGTCAT-3′ (anti-sense).

### 2.6 MTT assay

Transfected U251 and A172 cells were seeded into 96-well plate (Corning) and incubated for 12, 24, 48, and 72 h. The cell viability was determined by 3-(4, 5-dimethylthiazol-2-yl)-2,5-diphenyltetrazolium bromide (MTT; Sigma-Aldrich) staining. A total of 5 mg/mL MTT was added to each well, and the cultures were incubated for another 4 h at 37°C. The supernatant was aspirated, and formazan crystals were dissolved in 150 µL DMSO (Sigma-Aldrich). The spectrophotometric absorbance of each sample was measured at 450 nm. The experiments were conducted at least three times. For 50% inhibitory concentration (IC50) determination, transfected U251 and A172 cells were treated with various concentrations of TMZ (0–480 µM) for 48 h before the MTT assay.

### 2.7 Colony formation assay

Transfected U251 and A172 cells were seeded in 6-well plate and treated with TMZ for 14 days. The medium containing 60 µM of TMZ was refreshed every 3 days. After discarding the culture medium on day 14, the cells were fixed with 70% ethanol and stained with a 0.2% crystal violet. The cells were observed under a light microscope, and colonies containing more than 50 cells were counted. Three independent experiments were conducted.

### 2.8 Transwell assay

Cell invasion and migration were measured using Transwell chamber (8 µm pore size, Corning) coated with matrigel (Bection Dickinson, Franklin Lakes, USA) or uncoated. Transfected U251 and A172 cells were resuspended in 200 µL serum-free medium and then transferred in the upper chambers. The medium containing 10% FBS was used as a chemoattractant and loaded in the low chamber. The Transwell system was kept at 37°C for 24 h. The cells on the lower surface were stained with 0.1% crystal violet for 15 min at room temperature, followed with being photographed and counted. Three independent experiments were carried out.

### 2.9 Western blotting

Total protein from cultured U251 and A172 cells was isolated in RIPA lysis buffer (Beyotime) supplemented with cocktail protease inhibitor (Roche). The protein concentrations were determined by Bradford protein assay reagent (Bio-Rad, Shanghai, China). Equal amounts of protein (20 µg) from each sample were loaded for the standard procedures of the western blot assay. β-Actin on the same membrane was an internal standard to normalize protein levels. The primary antibodies were purchased from Cell Signaling Technology (CST; Danvers, Massachusetts, USA): AQP4 (#59678, 1:1,000) and β-actin (#5125, 1:1,000).

### 2.10 Luciferase reporter assay

Human LINC00461 wild-type (LINC00461 WT) and AQP4 3′ UTR fragment wild-type (AQP4 WT) containing the potential binding sites of hsa-miR-216a were cloned by PCR methods into psi-CHECK vector (Invitrogen), as well as the mutant types. U251 and A172 cells were co-transfected with miR-216a/NC mimics and either LINC00461 WT/MUT or AQP4 WT/MUT. After 48 h incubation, cells were collected to measure Firefly and Renilla luciferase activity using the dual-luciferase reporter assay system (Promega). All the data were the average of at least three independent transfections.

### 2.11 Xenograft experiment

A total of 6 NCG (NSG-like immune deficient) mice were purchased from the Model Animal Research Center at Nanjing University (Nanjing, China). The U251 cells (5 × 10^5^) stably transfected with sh-LINC00461 or sh-NC were subcutaneously injected into the posterior flank of the NCG mice (*n* = 3). The size of neoplasms was measured every 7 days after implantation, and the tumor volume was calculated with the formula: (length × width^2^)/2. On day 35, the mice were sacrificed, and the tumors were excised and weighed. The tumor tissues were stored at −80°C for total RNA and protein isolation.


**Ethical approval:** The research related to animal use has been complied with all the relevant national regulations and institutional policies for the care and use of animals. The animal experiments were performed under a standard protocol approved by the ethical standards of the institution of The Affiliated Mindong Hospital of Fujian Medical University and in accordance with the Guide for the Care and Use of Laboratory Animals.

### 2.12 Statistical analysis

Statistics were analyzed by SPSS 21.0 (SPSS Inc) and presented as the mean ± standard error. Student’s *t*-test method was utilized for comparison between two groups. The one-way analysis of variance was used for data comparison in multiple groups. *P* < 0.05 was considered as statistically significant.

## 3 Results

### 3.1 Expression of LINC00461 was upregulated in glioma patients

**Figure 1:**
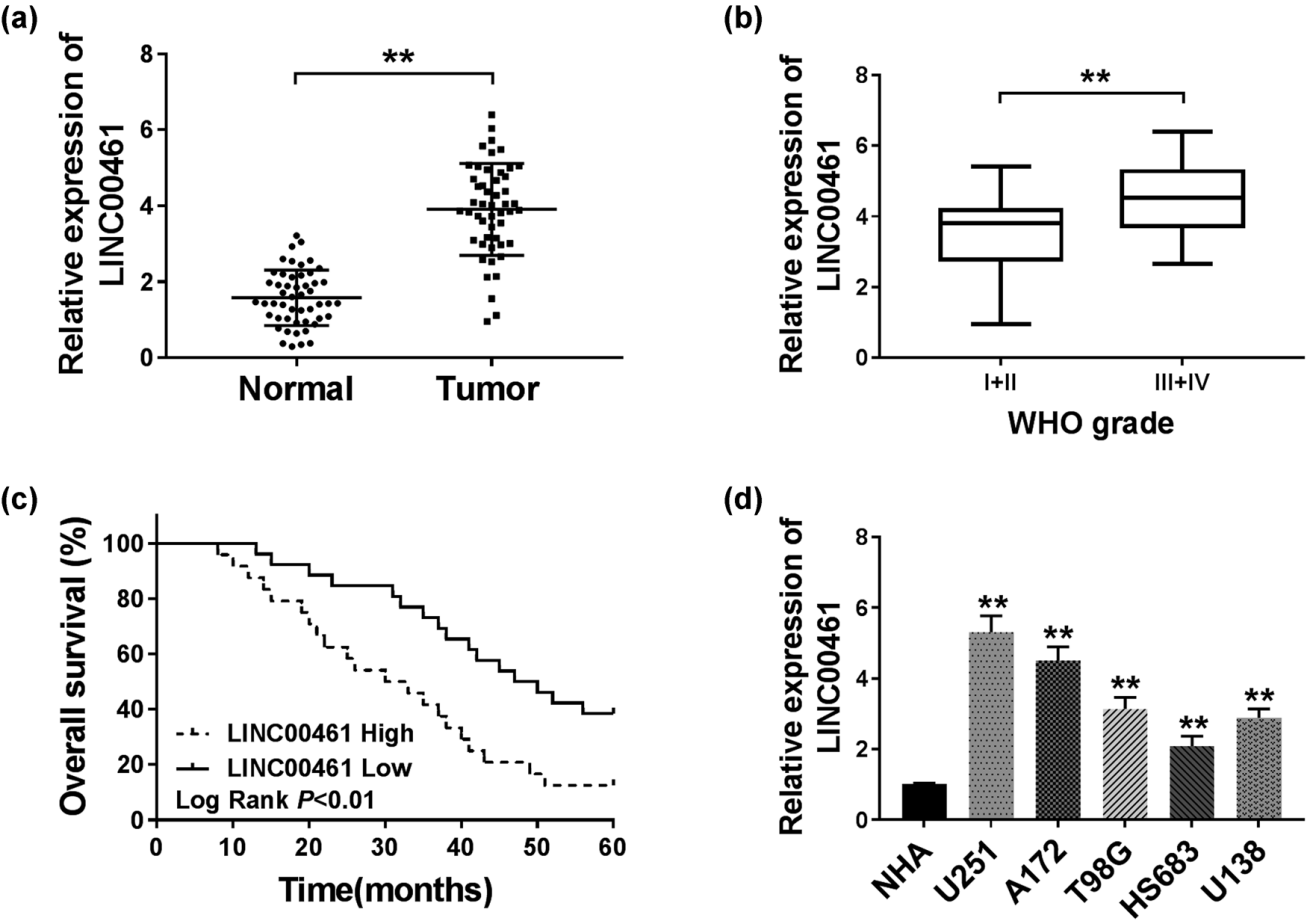
LncRNA LINC00461 (LINC00461) was highly expressed in glioma tissues and cells. (a) RT-qPCR detected LINC00461 levels in 50 paired glioma tissues and adjacent peritumoral brain edema tissues (normal). (b) RT-qPCR detected LINC00461 levels in these cases with different tumor node metastasis (TNM) stages: I + II, *n* = 11 and III + IV, *n* = 39. (c) The overall survival rate was recorded using Kaplan–Meier analysis in 50 glioma patients with high (*n* = 24) and low (*n* = 26) expression of LINC00261 after gross total resection followed by standard-of-care radiation therapy and chemotherapy. (d) RT-qPCR detected LINC00461 levels in glioma cell lines U251, A172, T98G, HS683, and U138 and normal cell line NHA. **p < 0.01.

To investigate whether LINC00461 participated in the progression of glioma, we first clarified its expression in patients with glioma. As shown in Figure 1a, the level of LINC00461 was extremely higher (more than fourfold, *p* < 0.01) in 50 glioma tissues than that in paired adjacent peritumoral brain edema tissues. Even higher level of LINC00461 was observed in advanced tumor grades (III + IV, *n* = 39; Figure 1b). Moreover, we divided these glioma patients into two groups, high expression of LINC00461 and low expression of LINC00461 according to the mean of LINC00461 level (Table 1), and found that high LINC00461 expression was correlated with larger tumor size, higher WHO grade, recurrence, and shorter cumulative 5-year survival rate (Figure 1c). Besides, we further confirmed its upregulation in glioma cell lines U251, A172, T98G, HS683, and U138 compared with normal cell line NHA (Figure 1d). These data showed the abnormal expression of LINC00461 in glioma tissues and cells and suggested its potential role in the pathogenesis of glioma.

### 3.2 Knockdown of LINC00461 inhibited glioma cell proliferation, migration, invasion, and chemoresistance *in vitro*

**Figure 2:**
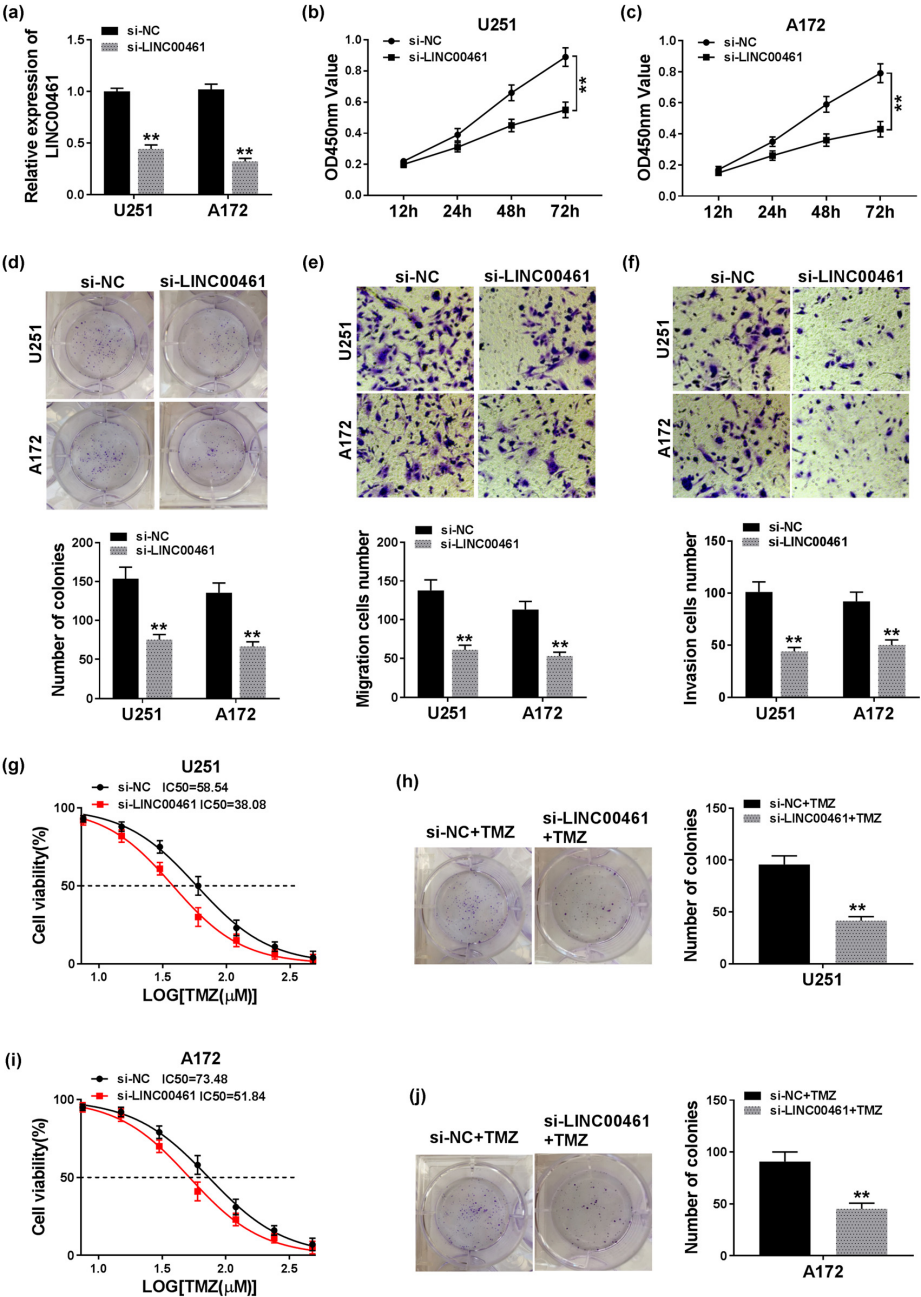
Knockdown of LINC00461 inhibited glioma cell proliferation, migration, invasion, and chemoresistance *in vitro*. U251 and A172 cells were transfected with siRNAs against LINC00461 (si-LINC00461) and its negative control (si-NC). (a) RT-qPCR detected LINC00461 levels at 24 h. (b and c) Cell viability was assessed using the MTT assay at 12, 24, 48, and 72 h. (d) Cloning ability was evaluated by the colony formation assay. (e and f) Transwell assays measured the ability of cell migration and invasion. The relative quantification of migratory cells and invasive cells was conducted. (g–j) The transfected U251 and A172 cells were treated with different doses of TMZ (1.0–2.5 µM, logarithm of concentration). Cell proliferation was determined by (g and i) MTT assay with 50% inhibitory concentration (IC50) of TMZ after TMZ (0, 7.5, 15, 30, 60, 120, 240, and 480 µM) treatment for 48 h, and (h and j) colony formation assay with cloning ability after 60 µM of TMZ treatment for 14 days. **p < 0.01.

To explore the role of LINC00461 in glioma, we selected U251 and A172 cells for further loss-of-function experiments due to the highest level of LINC00461 in these cells. First, U251 and A172 cells were transfected with si-LINC00461 or si-NC. The high transfection efficiency was determined by RT-qPCR, and si-LINC00461 could significantly knock down LINC00461 expression in both U251 and A172 cells (Figure 2a). The MTT assay illuminated that the cell viability was distinctively decreased after si-LINC00461 transfection for 48–72 h compared to si-NC transfection (Figure 2b and c). The cloning ability was evaluated by the colony formation assay, and the colony numbers were reduced during LINC00461 knockdown (Figure 2d). Transwell assays depicted the abilities of cell migration and invasion in U251 and A172 cells, which were both attenuated after silenced expression of LINC00461, as evidenced by the loss of migratory cells and invasive cells (Figure 2e and f). TMZ is a classic chemotherapeutic drug that is widely used as treatment for glioma. With TMZ treatment, IC50 values were significantly declined in U251 and A172 cells transfected with si-LINC00461 (Figure 2g and i), and LINC00461 silencing also depressed the cloning ability of TMZ-treated U251 and A172 cells (Figure 2h and j). These results showed that LINC00461 knockdown could inhibit glioma cell proliferation, migration, invasion, and TMZ resistance *in vitro*.

### 3.3 LINC00461 negatively regulated miR-216a through acting as a ceRNA

**Figure 3:**
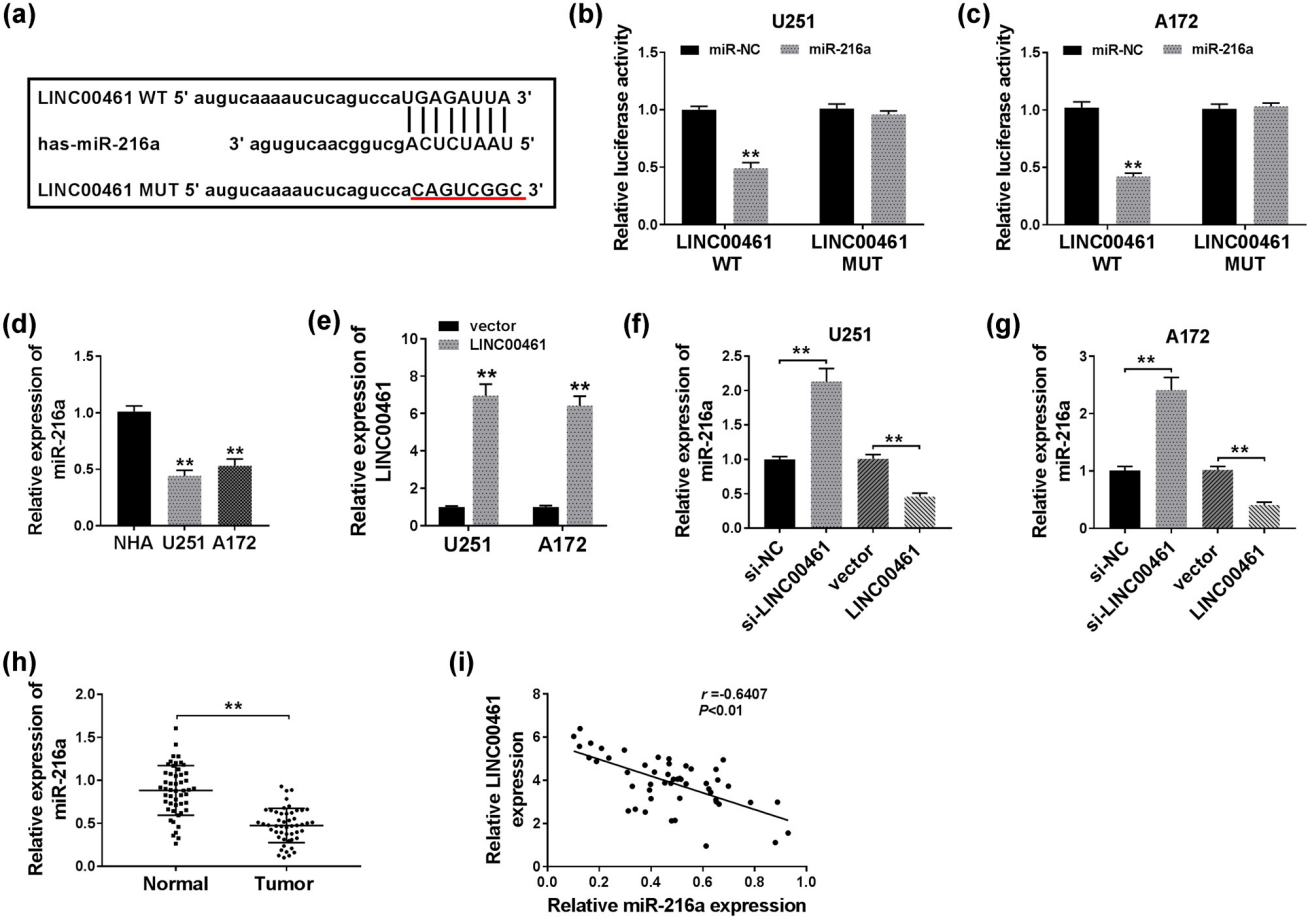
Identification of the negative regulatory relationship of LINC00461 on miRNA-216a (miR-216a) via target binding. (a) The predicted miR-216a binding sites in *LINC00461* gene according to miRcode. The corresponding sequence in the mutated version was also shown. (b and c) The luciferase activity of LINC00461 wild-type (LINC00461 WT) in U251 and A172 cells transfected with miR-216a mimics or its negative control (miR-NC mimics). (d) RT-qPCR detected miR-216a levels in glioma cell lines U251 and A172 and normal cell line NHA. (e) RT-qPCR detected LINC00461 levels in U251 and A172 cells transfected with pcDNA-LINC00461 (LINC00461) or their negative controls (vector). (f and g) Expression levels of miR-216a in U251 and A172 cells transfected with si-LINC00461, LINC00461, or their negative controls. (h) RT-qPCR detected miR-216a levels in 50 paired glioma tissues and adjacent nonneoplastic tissues. (i) Analysis of the correlation between expressions of LINC00461 and miR-216a in glioma tissues (Spearman correlation analysis). **p < 0.01.

The putative downstream target genes of LINC00461 were investigated on miRcode website (http://www.mircode.org). We noticed a novel potential target relationship between LINC00461 and miR-216a, and the complementary binding site was presented in Figure 3a. To further confirm that, the luciferase reporter assay showed that the luciferase activity of psiCHECK-2 vector containing LINC00461 WT was remarkably reduced in U251 and A172 cells after co-transfection with miR-216a mimics compared to miR-NC mimics transfection (Figure 3b and c); however, there was no difference in LINC00461 MUT groups. Besides, miR-216a levels were lower in glioma cell lines U251 and A172 than normal cell line NHA (Figure 3d). Overexpression vector pcDNA-LINC00461 resulted in dramatically high expression of LINC00461 in U251 and A172 cells (Figure 3e). Meanwhile, miR-216a expression was negatively regulated by LINC00461 (Figure 3f and g). In glioma patients, the expression of miR-216a was dramatically downregulated in glioma tissues (Figure 3h) in a LINC00461-correlated manner (Figure 3h and i). These results showed that LINC00461 negatively regulated miR-216a by targeted binding.

### 3.4 AQP4 was targeted and downregulated by miR-216a

**Figure 4:**
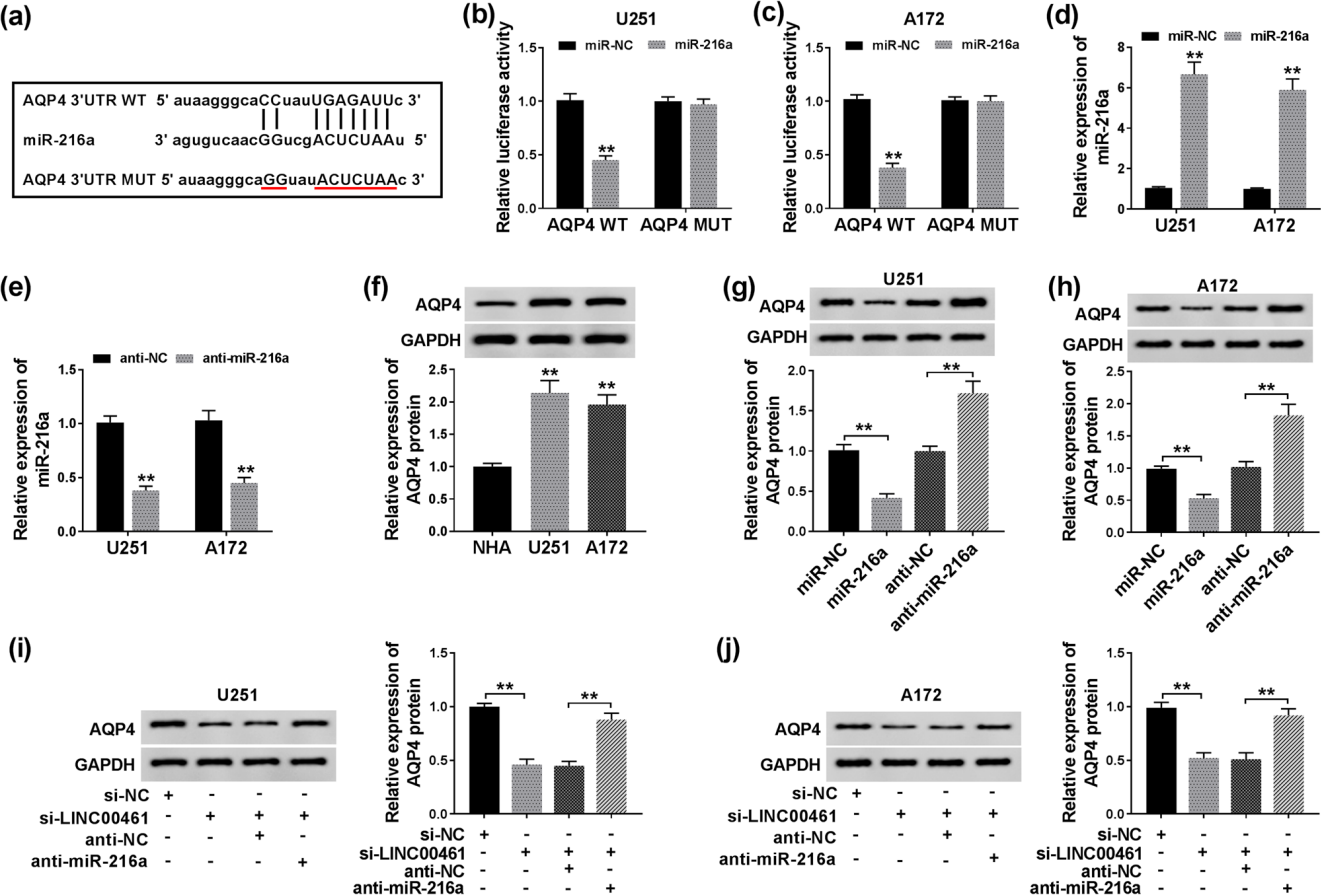
AQP4 was targeted and downregulated by miR-216a. (a) The predicted hsa-miR-216a binding sites in the 3′ UTR of AQP4 gene according to the microRNA.org database. The corresponding sequence in the mutated version was also shown. (b and c) Luciferase activity of AQP4 3′ UTR wild-type (AQP4 WT) in U251 and A172 cells transfected with miR-216a mimics (miR-216) or miR-NC mimics (miR-NC). (d and e) RT-qPCR detected miR-216a levels in U251 and A172 cells transfected with miR-216, miR-NC, and miR-216a inhibitors (anti-miR-216) or miR-NC inhibitors (anti-NC). Western blotting examined AQP4 protein expression level in (f) U251, A172, and NHA cells, (g and h) U251 and A172 cells transfected with miR-216a, anti-miR-216a, or their negative controls, and (i and j) U251 and A172 cells transfected with si-NC alone, si-LINC00461 alone or together with either anti-miR-216a or anti-NC. **p < 0.01.

The putative downstream target genes of miR-216a were investigated on microRNA.org website (http://www.microrna.org/microrna/getDownloads.do). We observed a novel potential target relationship between miR-216a and AQP4, and the complementary binding site is shown in Figure 4a. The luciferase reporter assay also confirmed the potential binding between miR-216a and AQP4 (Figure 4b and c). In addition, AQP4 was dramatically upregulated in glioma cell lines U251 and A172 than normal cell line NHA (Figure 4f). When miR-216a expression in U251 and A172 cells was forcedly overexpressed or silenced by transfection of miR-216a mimics or anti-miR-216a (Figure 4d and e), AQP4 protein expression was negatively modulated by miR-216a (Figure 4g and h). Moreover, downregulation of miR-216a could abolish the inhibitory effect of LINC00461 knockdown on AQP4 expression (Figure 4i and j). These results showed that AQP4 was negatively regulated by miR-216a through target binding.

### 3.5 Downregulation of AQP4 mediated the suppressive role of LINC00461 knockdown in glioma cells *in vitro* via miR-216a

**Figure 5:**
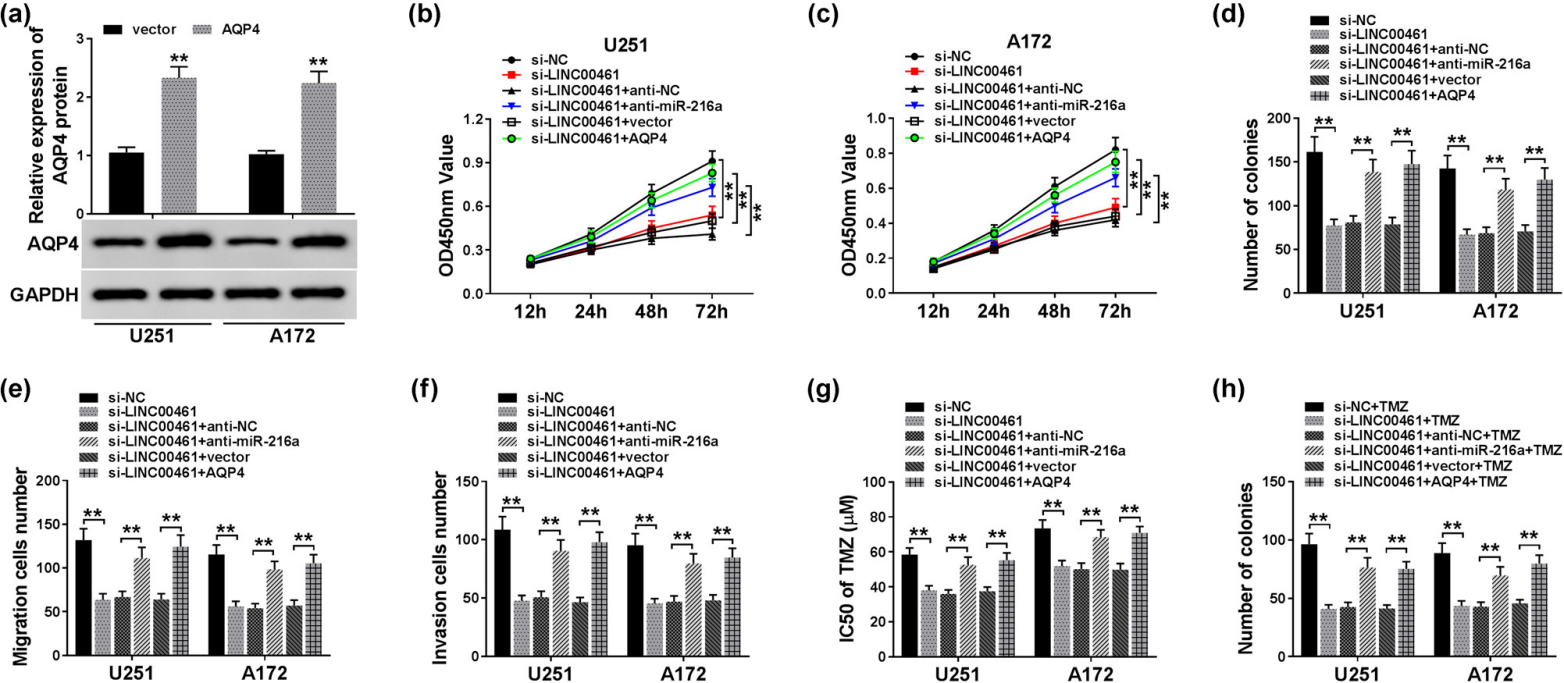
Effect of AQP4 expression on the role of LINC00461/miR-216a axis in glioma cells *in vitro*. (a) Western blotting examined AQP4 protein level in U251 and A172 cells transfected with pcDNA-AQP4 (AQP4) or vector for 24 h. (b–h) U251 and A172 cells were transfected with si-LINC00461 or si-NC and co-transfected with si-LINC00461 and anti-miR-216a or anti-NC and co-transfected with si-LINC00461 and siRNA against AQP4 (si-AQP4) or si-NC. After transfection, cell proliferation, migration, and invasion were measured by (b and c) MTT assay with cell viability, (d) colony formation assay for cloning ability, and (e and f) Transwell assays for cell migration and invasion abilities. (g and h) MTT assay and colony formation assay were used to assess TMZ sensitivity with IC50 values and cloning ability. **p < 0.01.

In consideration of the regulatory effect of LINC00461/miR-216a axis on AQP4 expression, we wondered whether different expression of AQP4 could affect its role in glioma cells. Therefore, ectopic expression of AQP4 was fulfilled by transfection of pcDNA-AQP4 (Figure 5a). Then, U251 and A172 cells were transfected with si-LINC00461 alone or together with anti-miR-216a or pcDNA-AQP4. As shown in Figure 5b and c, LINC00461 knockdown suppressed glioma progression, which was counteracted in the presence of anti-miR-216a or pcDNA-AQP4, as evidenced by rescued cell viability and cloning ability (Figure 5b–d) and improved migration and invasion (Figure 5e and f). In terms of TMZ resistance, IC50 value and cloning ability of U251 and A172 cells were poor when LINC00461 silenced, which was further improved by miR-216a deletion or AQP4 upregulation (Figure 5g and h). These data demonstrated that LINC00461 knockdown suppressed glioma cell development *in vitro* through upregulating miR-216a and downregulating AQP4, suggesting a LINC00461/miR-216a/AQP4 pathway in glioma cells.

### 3.6 Knockdown of LINC00461 restricted tumor growth of glioma cells *in vivo*

**Figure 6:**
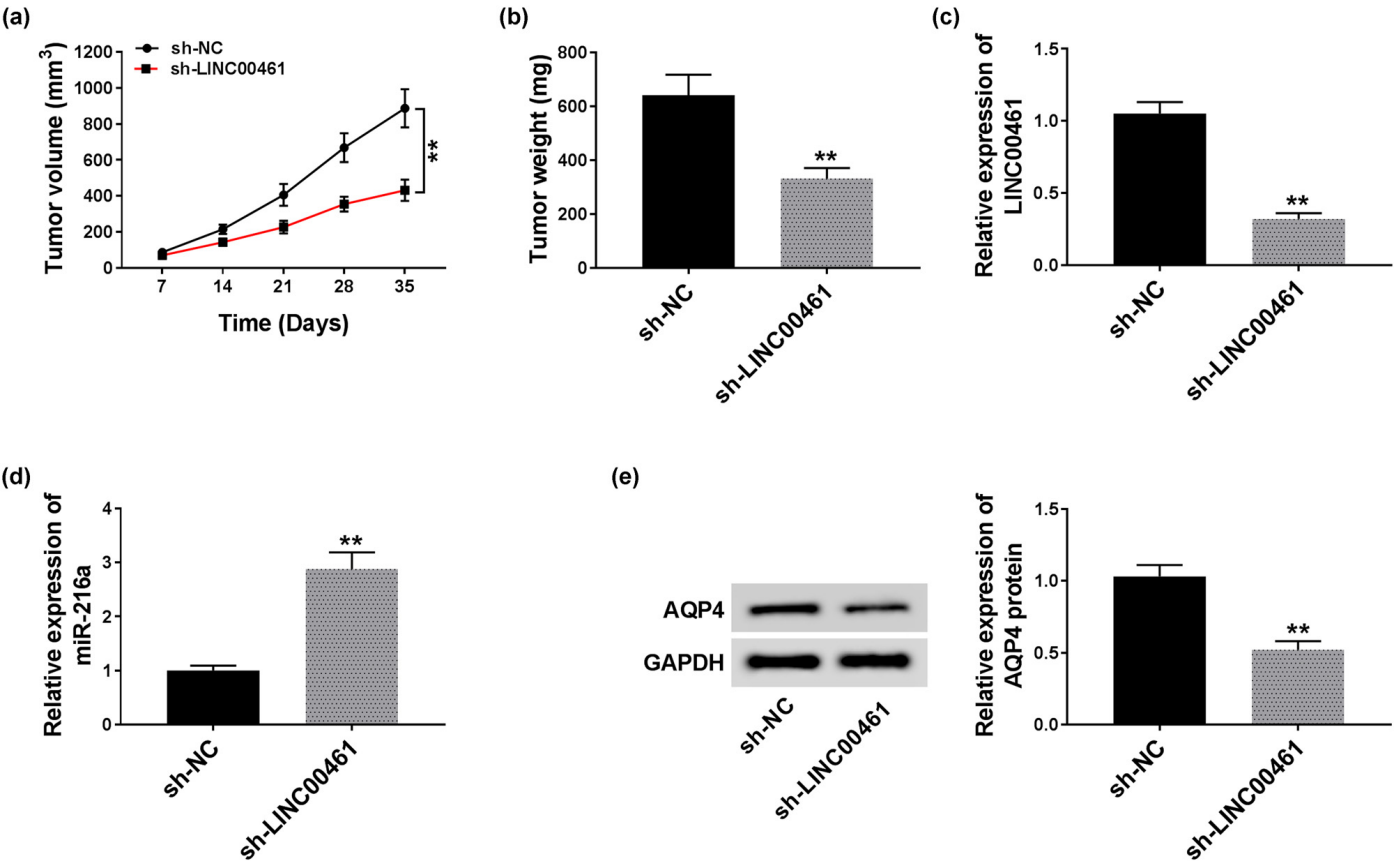
Knockdown of LINC00461 restricted the tumor growth of glioma cells *in vivo*. U251 cells stably transfected with sh-NC or sh-LINC00461 were subcutaneously injected into the posterior flank of the NCG mice (*n* = 3). (a) Tumor volumes were calculated depending on the sizes of neoplasms. (b) Tumor weight was measured after implantation for 35 days. (c–e) Expression of LINC00461, miR-216a, and AQP4 was measured by RT-qPCR and western blotting. **p < 0.01.

To investigate the tumorigenesis *in vivo*, U251 cells were stably transfected with sh-NC or sh-LINC00461, followed with subcutaneous injection into the posterior flank of the NCG mice (*n* = 3). The tumor volume and the tumor weight data indicated a suppressed tumor growth of LINC00461-downregulated U251 cells in mice (Figure 6a and b). At the same time, expression of LINC00461 and AQP4 decreased in xenograft tumor tissues in sh-LINC00461 group, whereas miR-216a was higher (Figure 6c–e). These outcomes showed a suppressive role of LINC00461 knockdown in tumor growth *in vivo* via miR-216a and AQP4.

## 4 Discussion

Downregulated and upregulated lncRNAs affect glioma cell proliferation, migration, and invasion, which are the major characteristics of cancer cells. For example, lncRNA HOTAIR was upregulated in glioma patients and promoted glioma progression through several mechanisms such as acting as ceRNA for miRNA-126-5p and miRNA-326 [13,14], enhancing angiogenesis [15], regulating an 18-gene cell cycle-related mRNA network [16], and activating PI3K/AKT and MEK1/2 pathways [14]. LncRNA TUG1 was downregulated in glioma tissues and cells and inhibited cell proliferation and invasion by targeting miRNA-145 in regulating glioma cell self-renewal [17], sponging miRNA-299 in promoting angiogenesis [18], and interacting with miR-144 in modulating blood–tumor barrier permeability [19]. In this study, we observed extremely high expression of LINC00461 in glioma tissues and cell lines U251, A172, T98G, HS683, and U138. Knockdown of LINC00461 suppressed glioma cell proliferation, migration, invasion, and TMZ resistance in U251 and A172 cells *in vitro* and inhibited tumor growth *in vivo* through targeting miR-216a and downregulating AQP4.

LINC00461 was first identified as a novel intergenic lncRNA, termed evolutionary conserved and expressed in neural tissues (ECONEXIN) that functioned in a glioma mouse model [12]. ECONEXIN was dominantly located in the cytoplasm of U87 and U251 cells, and its inhibition led to decreased cell proliferation by targeting miRNA-411-5p/topoisomerase 2 alpha (TOP2A) axis. Later, Yang et al. [8] demonstrated that LINC00461 is important for glioma progression as evidenced by declined cyclinD1/A/E expression, G0/G1 cell cycle arrest, inhibited glioma cell proliferation, migration, and invasion when LINC00461 was downregulated, which was accompanied with inhibition of miRNA-9; meanwhile, MAPK/ERK and PI3K/AKT signaling pathways were inactivated, while cell apoptosis did not alter in U251 and U87MG cells after lentivirus infected with shRNA against LINC00461. Here, we supported the suggestion that LINC00461 knockdown suppressed glioma cell proliferation, migration, and invasion *in vitro*, which was inconsistent with the previous study [8]. Moreover, we confirmed a promoting effect of LINC00461 silencing on the TMZ sensitivity. In clinic, it showed a positive correlation between LINC00461 high expression and tumor size, WHO grade, recurrence, and poor prognosis. LINC00461/miR-216a/AQP4 pathway could contribute to tumorigenesis of glioma. However, the involved signaling pathways such as MAPK/ERK and PI3K/AKT pathways remain to be further detected in glioma cells.

To date, the ceRNA network of LINC00461 in cancers has been established. It has been reported that LINC00461/miRNA-149-5p/LRIG2 existed in hepatocellular carcinoma [9], LINC00461/miRNA-15a/miRNA-16/Bcl-2 happened in multiple myeloma [10], LINC00461/miRNA-30a-5p/integrin β3 occurred in breast cancer [11], and LINC00461/miRNA-411-5p/TOP2A participated in glioma [12]. Therefore, we aimed to investigate a novel miRNA/mRNA axis targeted by LINC00461 in glioma.

In the present study, we noticed a putative binding site of miR-216a in LINC00461 according to the miRcode database, and the luciferase reporter assay further verified this potential target relationship. In addition, miR-216a expression was negatively regulated by LINC00461 in glioma tissues and cell lines U251 and A172 cells, and downregulation of miR-216a could abolish the suppressive role of LINC00461 knockdown in cell proliferation, migration, invasion, and apoptosis inhibition *in vitro*. Hence, we suggested LINC00461/miR-216a axis in glioma. Previous studies have illuminated the important role of miR-216a in gliogenesis. For example, Zhang et al. [20] found that miR-216a was significantly decreased in glioma tissues and cell line including U251MG, U87MG, U118, and A172, and its overexpression could suppress the proliferation, migration, and invasion of glioma cells by targeting Leucine-rich repeat-containing G protein-coupled receptor 5 (LGR5), which suggests a tumor-suppressive role of miR-216a in glioma. Very recently, emerging studies indicated that lncRNA DANCR [21] and GHET1 [22] were sponges of miR-216a in glioma cells in regulating cell progression and angiogenesis. These studies together might imply miR-216a as a potential therapeutic target for glioma treatment, and this hypothesis remains to be further proved.

AQP4 is the most important AQP family member [23]. Originally, AQP4 was well descripted as brain-specific regulator; recently, there has emerged a surprising link between AQPs including AQP4 and tumors [24]. Several cell progressions such as proliferation, migration, and angiogenesis are abnormal during AQP4 dysregulation. In cerebral ischemic injury, AQP4 not only took part in oxygen–glucose deprivation (OGD)–induced injury in primary cultured astrocytes but also mediated miRNAs miRNA-29a [25], miRNA-145 [26], and miRNA-320a [27] effect on LDH release, apoptosis, and cell health *in vitro* and infarct volume *in vivo*. In glioma, differential expression of AQP4 was pointed out in human glioma tissues [28], and AQP4 was upregulated in glioma-associated edema [29]. AQP4 was significantly upregulated in glioblastoma multiforme (IV stage) compared to low-grade glioma [30]. The close involvement of AQP4 in cell migration and invasion was summarized in glioma malignancy, as well as drug resistance [31]. However, the association between miRNAs and AQP4 remains unclear in glioma except one. Xiong et al. [32] declared that miRNA-320a/AQP4 axis inhibited cell migration and invasion in U87 and U251 cells. In the present study, we identified that AQP4 served as a downstream target for miR-216a and confirmed that LINC00461/miR-216a axis regulated glioma cell proliferation, apoptosis, migration, and invasion depending on modulating AQP4 expression. These results provide a novel, vital miRNAs/AQP4 axis in glioma.

TMZ resistance always occurs in glioma patients during its treatment. Then, promoting the sensitivity of TMZ to glioma cells would be a promising approach, and the exact molecular mechanism in that is urgent to uncover. Many lncRNAs had been elaborately discussed in this chemoresistance in glioma, such as LINC01198, SNHG15, and MALAT1 [33,34,35]. Here, we declared a novel lncRNA involved in TMZ resistance through the well-known ceRNA network in glioma. Moreover, we provided the first evidence of the target relationship between miR-216a and either LINC00461 or AQP4. The results suggested a novel miR-216a/AQP4 axis underlying LINC00461 in regulating glioma cell progression. However, the role of LINC00461/miR-216a/AQP4 pathway in glioma cell processes such as angiogenesis remains to be further uncovered, as well as its involved signaling pathways such as PI3K/AKT [21], JAK2/STAT3, and p53/survivin [22].

## 5 Conclusion

In conclusion, this study supported the tumor-promoting role of LINC00461 in glioma. The results demonstrated that the knockdown of LINC00461 contributed to TMZ sensitivity and inhibited cell proliferation, migration, and invasion in U251 and A172 cells through targeting miR-216a and downregulating AQP4. Our work suggested LINC00461/miR-216a/AQP4 circuit as a novel pathway in the occurrence and development of glioma.
